# Direct Bacterial Killing *In Vitro* by Recombinant Nod2 Is Compromised by Crohn's Disease-Associated Mutations

**DOI:** 10.1371/journal.pone.0010915

**Published:** 2010-06-01

**Authors:** Laurent-Herve Perez, Matt Butler, Tammy Creasey, JoAnn Dzink-Fox, John Gounarides, Stephanie Petit, Anna Ropenga, Neil Ryder, Kathryn Smith, Philip Smith, Scott J. Parkinson

**Affiliations:** 1 Gastrointestinal Disease Area, Novartis Institutes for Biomedical Research, Horsham, United Kingdom; 2 Analytical Sciences, Novartis Institutes for Biomedical Research, Cambridge, Massachusetts, United States of America; 3 Infectious Disease Area, Novartis Institutes for Biomedical Research, Cambridge, Massachusetts, United States of America; University of Hyderabad, India

## Abstract

**Background:**

A homeostatic relationship with the intestinal microflora is increasingly appreciated as essential for human health and wellbeing. Mutations in the leucine-rich repeat (LRR) domain of Nod2, a bacterial recognition protein, are associated with development of the inflammatory bowel disorder, Crohn's disease. We investigated the molecular mechanisms underlying disruption of intestinal symbiosis in patients carrying Nod2 mutations.

**Methodology/Principal Findings:**

In this study, using purified recombinant LRR domains, we demonstrate that Nod2 is a direct antimicrobial agent and this activity is generally deficient in proteins carrying Crohn's-associated mutations. Wild-type, but not Crohn's-associated, Nod2 LRR domains directly interacted with bacteria *in vitro*, altered their metabolism and disrupted the integrity of the plasma membrane. Antibiotic activity was also expressed by the LRR domains of Nod1 and other pattern recognition receptors suggesting that the LRR domain is a conserved anti-microbial motif supporting innate cellular immunity.

**Conclusions/Significance:**

The lack of anti-bacterial activity demonstrated with Crohn's-associated Nod2 mutations *in vitro*, supports the hypothesis that a deficiency in direct bacterial killing contributes to the association of Nod2 polymorphisms with the disease.

## Introduction

Crohn's disease is a chronic relapsing inflammatory bowel disorder characterised by transmural and discontinuous lesions of the intestinal tract [1],[2]. It is generally accepted that the characteristic gut inflammation of Crohn's (as well as ulcerative colitis) is the result of a robust immune response against commensal bacteria in the gastrointestinal tract. Currently, it is not clear what underlies this loss of tolerance, however patients demonstrate increased levels of attaching/effacing and intracellular bacteria consistent with an initiating role for bacteria in the pathogenesis of the disease [3]. Environmental factors (such as smoking) as well as genetic factors also play a role in disease pathogenesis [4]. Genome-wide linkage studies of Crohn's disease families initially identified a susceptibility locus on chromosome 16 [5]. This linkage was subsequently confirmed as being a result of mutations in the intracellular pattern recognition receptor (PRR) Nod2 [6]. Three distinct Nod2 single nucleotide polymorphisms (SNPs) have been associated with increased risk of developing Crohn's disease. All three are coding mutations contained within (G908R, 3020insC), or adjacent to (R702W), the LRR domain at the C-terminus of the protein. LRR domains are common to many PRRs and are believed to confer sensitivity to pathogen-associated molecular patterns (PAMPs).

## Results

**Figure 1:**
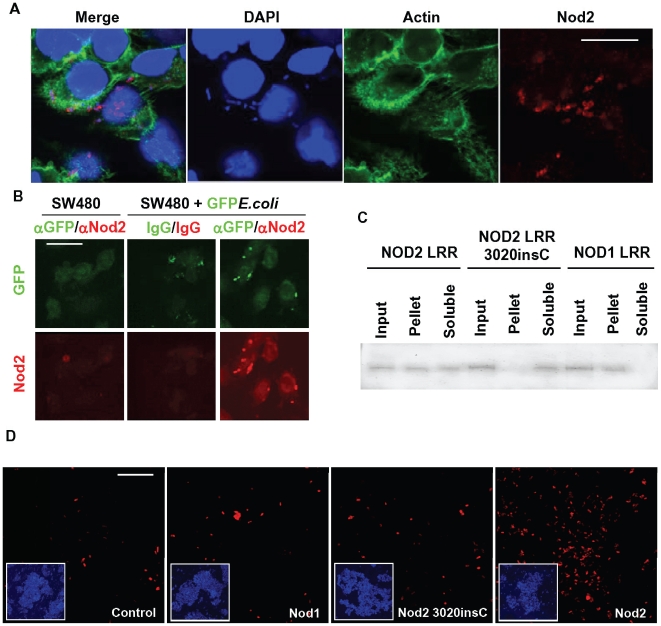
Nod2, but not the Crohn's-associated 3020insC mutation, directly associates with bacteria. **A**, SW480 intestinal epithelial cells were used to investigate the distribution of endogenous Nod2 protein following inoculation of the cells with *E.coli* (ATCC 25922) at an MOI of 1000∶1. Cells were processed for immunofluorescence using a polyclonal antibody raised against the LRR domain of Nod2, immunopurified on the antigen and further processed on an *E.coli* lysate column to remove contaminating anti-bacterial antibodies. Controls are shown in Figure S1 demonstrating specificity of the Nod2 antibody. Bar = 15 µm. **B**, *E.coli* expressing GFP were generated by transformation of ATCC 25922 with a cDNA encoding GFP in pUC19. SW480 cells were treated as described in Figure 1a. An antibody against GFP was used to enhance the GFP signal although the fluorescent protein was generally visible (centre top panel). Isotype antibody control for GFP (chicken IgY) and rabbit IgG processed on an *E.coli* lysate column were used as controls (centre panels). Bar = 30 µm. **C**, Association of His-tagged recombinant protein derived from cDNAs encoding Nod2 LRR, Nod2 3020insC LRR and Nod1 LRR domains with *E.coli*. 10 ng of each protein was added to approximately 10^6^ bacteria in 100 µl of LB media, incubated for 30 minutes at room temperature, and the samples centrifuged at low speed to collect the bacteria and associated proteins. The bacterial pellet and supernatants were reconstituted in equal volumes and loaded on an SDS-PAGE gel. Following transfer to nitrocellulose, the samples were analysed by Western blot using an anti-His antibody (Sigma). **D**, Direct binding of Nod2, but not Nod2 3020insC, to *E.coli* in vitro. Recombinant Nod2, Nod2 3020insC or Nod1 LRR domains (35 µg/ml) were added to *E.coli* at 37°C overnight. Bacteria were collected by centrifugation, washed and the association of LRR domains determined by immunofluorescent labelling of the bacteria with anti-Nod2 antibody (described in Figure 1a) and goat anti-rabbit 2° antibody. A sample of the labelled bacteria were placed on a sealed coverslip and analysed by fluorescent microscopy. Insets demonstrate the presence of comparable numbers of bacteria in each of the images as demonstrated by DAPI staining. Bar = 10 µm.

Unlike *H. pylori's* part in the pathogenesis of stomach ulcers, a single pathogen associated with development of Crohn's disease has not been demonstrated although several have been proposed [7]. Due to their constant exposure to the microbiota of the gastrointestinal tract, epithelial cells are the primary point of contact with the commensal flora. In order to investigate the mechanism by which Nod2 protects the gastrointestinal tract, polyclonal antibodies were raised against recombinant human Nod2 LRR domains and used to examine the localisation of endogenous Nod2 in an intestinal epithelial cell line incubated with a non-pathogenic *Escherichia coli* strain (Figure 1a). In the absence of bacteria, Nod2 was expressed at low levels and distributed throughout the cytosol (Figure S1). Following incubation with *E.coli* for 2 hours, Nod2 aggregated within the cytoplasm of the cells (Figure 1a, Figure S1). The observed Nod2-positive structures were consistent with the size and characteristic shape of *E.coli* and were co-stained with DAPI. The presence of these bacteria inside the exposed cells was surprising considering the strain used (FDA strain Seattle 1946 [DSM 1103, NCIB 12210, ATCC25922]) is a biosafety level 1 bacterium that, to our knowledge, has not demonstrated any previous evidence of pathogenic potential. We next generated GFP-expressing *E.coli* and repeated the experiment to confirm that Nod2 was recruited to bacteria-containing structures within the cell (Figure 1b). Nod2 is generally believed to be a sensor of muramyl dipeptide (MDP), a component of the bacterial proteoglycan coat. Until now, a direct interaction between Nod2 and bacteria has not been demonstrated. We tested this possibility *in vitro* and confirmed that recombinant Nod2 LRR domains associated directly with *E.coli* using two different assays for direct bacterial binding. Recombinant Nod2, Nod2 3020insC and Nod1 LRR domains were incubated with *E.coli,* collected by centrifugation, and the distribution of the Nod2 and Nod1-derived proteins in the bacterial pellet and/or supernatant determined by Western blot (Figure 1c). This assay demonstrated a significant accumulation of Nod2 LRR domains with the bacterial pellet. This association was not observed using the 3020insC LRR domain. Nod1 LRR also distributed with the bacterial pellet demonstrating that it too can directly recognise bacteria. In addition, purified LRR domains of Nod2 were associated with *E. coli* as demonstrated by staining of the bacteria with an antibody raised against the LRR domain of Nod2 (Figure 1d). Incubation of bacteria with Nod1 LRR domains did not give significant staining above background (Control) despite Nod1 LRR domain association with *E.coli* (Figure 1c) demonstrating the specificity of the antibody for Nod2. The LRR domains containing the 3020insC polymorphism could not be detected on the bacteria above background levels suggesting this Crohn's-associated polymorphism confers an inherent defect in bacterial recognition. Direct interaction of the Nod2 LRR domain with the gram-positive bacteria *E. faecalis* was also demonstrated (Figure S2).

**Figure 2:**
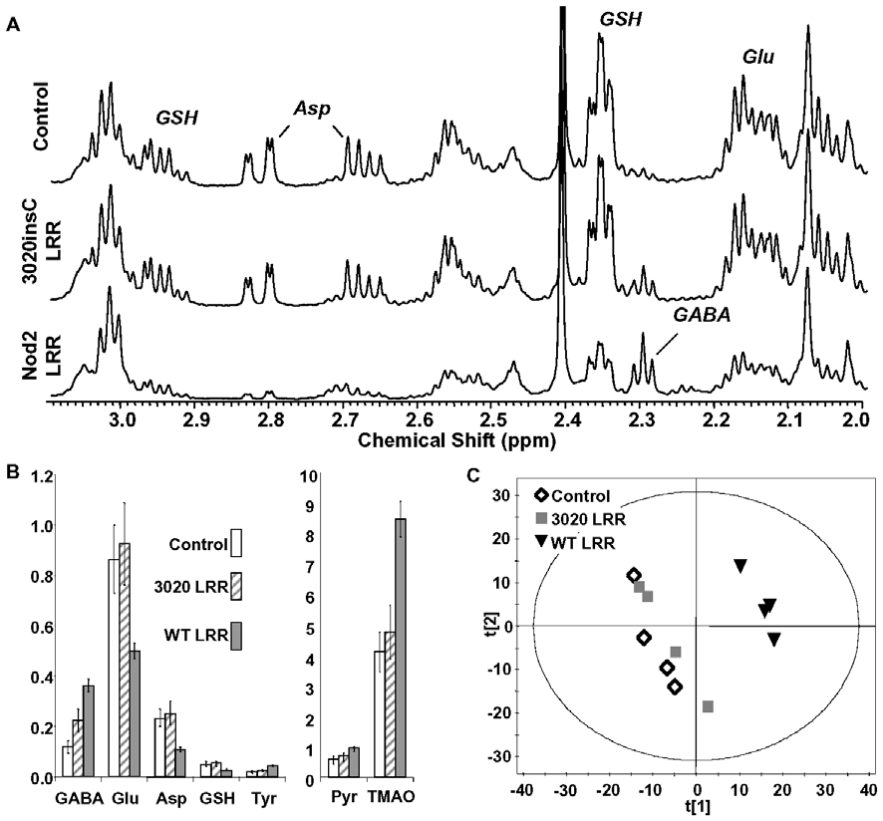
Nod2 LRR domains impact bacterial metabolism. **A**, Expansion of ^1^H-NMR spectrum (δ^1^H 3.1 to 2.0 ppm) of cell extracts from Control (BSA treated), Nod2 LRR and Nod2 3020insC LRR domains as indicated. **B**, Metabolic perturbation induced by Nod2 LRR and Nod2 3020incC LRR treatment in *E. Coli*. GABA; γ-aminobutyrate, Glu; glutamate, Asp; aspartate, GSH; glutathione, Tyr; tyrosine, Pyr; pyruvate, TMAO; trimethylamine oxide. All metabolite changes indicated with Nod2 LRR were statistically significant versus control *E.coli* (p<0.05, Student's T-test). The data are summarised in Table S1. **C**, Principle component analysis (PCA) score plot of *E.coli* metabolism treated as indicated with LRR domains from Nod2, Nod2 3020insC or BSA. PCA of the NMR data was performed using SIMCA-P v.10 (Umetrics AB, Umeå, Sweden). This data reduction method allows the visualization of the effect of treatment on the cellular metabolism, the clustering of groups of data is based solely on the similarity of the input spectral data. Each symbol represents the ^1^H-NMR spectrum of an individual cell extract. The axis, principal component 1 (t[1]) and 2 (t[2]), represent the top two most abundant correlated variation within the data set. The separation of the WT LRR from the Control and 3020incC LRR cells is due to specific perturbations in cellular metabolism.

We next investigated the consequences of the observed LRR interaction with bacteria. Following overnight treatment of various bacterial strains with Nod2 LRR domains, a pellet could be observed in the bottom of the treated, but not control tubes (not shown). Examination of the pellet by microscopy revealed aggregation of LRR-treated bacteria suggesting Nod2 LRR domains were directly influencing the phenotype of a broad range of bacteria (Figure S3). Therefore, we sought to identify and quantitate the impact of the Nod2 LRR domains on exposed bacteria. *E.coli* was cultured in LB media in the presence of the Nod2 LRR domains for 6 hours and the metabolic profile of the bacteria assessed. Metabolomic analysis revealed that Nod2 LRR domains induced significant and specific perturbations to cellular metabolism (Figure 2, Table S1). These included a 3-fold increase in γ-aminobutyrate, a 40–50% decrease in glutamate, aspartate and glutathione and a 2-fold increase in trimethylamine oxide (TMAO). The loss of glutathione would be expected to be detrimental to cell survival as glutathione is central to the regulation of intracellular K^+^ and detoxification of methylglyoxal in *E.coli* [8]. The loss of cellular glutamate and aspartate coupled with an increase in γ-aminobutyrate suggests the induction of amino acid decarboxylases consistent with cytoplasmic acidification [9]. LRR domains carrying the 3020insC did not induce similar perturbations to the cellular metabolite profile suggesting proteins with Crohn's-associated mutations cannot target bacterial metabolism due to their lack of direct binding to the bacteria (Figure 1).

**Figure 3:**
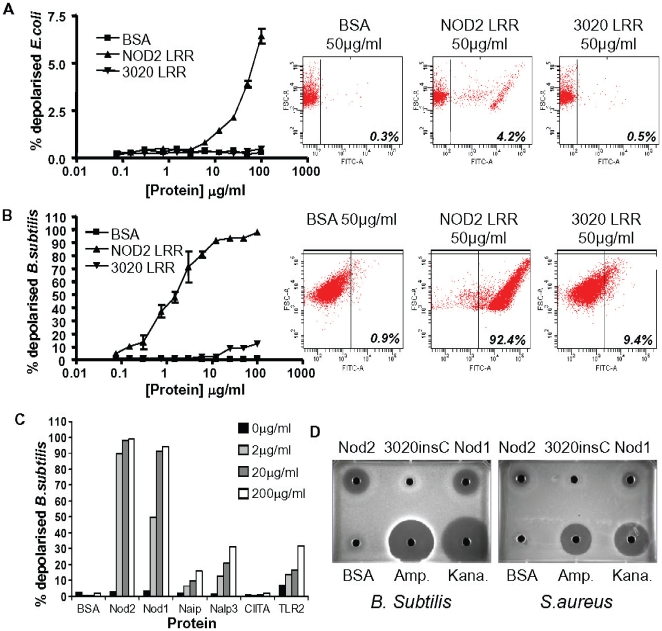
Bacterial killing by purified Nod2 LRR domains is deficient in the Crohn's-associated Nod2 3020insC mutant. Results shown are all representative of several experiments. **A,B**, Nod2 LRR domains influence the membrane polarity of *E.coli* and *B.subtilis*. Proteins were added at the concentration indicated to 5×10^5^ bacteria in 100 µL growth medium and incubated for 2 hours at 37°C. 15 minutes prior to the end of the time course, 50 µl of 10 µg/ml DiBAC4 solution was added to each well. Plates were washed twice with 750 µl ice cold PBS/well. The percentage of depolarised bacteria taking up the dye was determined by flow cytometry. **C**, Anti-bacterial activity of Nod1 and Nod2 but not Nod2 3020insC LRR domains demonstrated by agar diffusion assay. Agar plates were inoculated with a lawn of the indicated bacteria. Approximately 0.5 cm diameter holes were punched into the agar with a sterile glass pipette and the indicated protein (BSA protein control or indicated LRR domain) or antibiotic (ampicillin or kanamycin) added to each well at a concentration of 0.5 mg/ml in sterile PBS. **D**, *B. subtilis* membrane polarity is influenced by the LRR domains from a range of pattern-recognition receptors. Bacteria were treated with the indicated LRR domains as described for Figure 3a and their effect on the membrane polarity of the bacteria evaluated.

Previous reports have shown that Nod2 can protect cultured cells from bacterial infection and this activity is deficient in cells expressing the 3020insC polymorphism [10]. In addition, mice deficient in Nod2 (as well as mice carrying the equivalent mutation to the human 3020insC polymorphism) are more susceptible to infection by various bacteria [11]–[13]. Based on these reports and our own observations, we hypothesized that Nod2, directly binds to bacteria and kills them via the LRR domain. *E.coli* were incubated with increasing doses of the recombinant LRR domains of either Nod2 or Nod2 3020insC and the integrity of the bacterial membrane evaluated using a flow cytometric assay previously used to study the activity of anti-bacterial defensins [14]. Incubation with wild-type Nod2 LRR domain resulted in membrane depolarisation indicative of anti-bacterial activity (Figure 3a). This activity could not be observed with the Nod2 3020insC LRR domains. While *E.coli* were only partially susceptible to Nod2-induced membrane depolarisation, gram positive *B. subtilis* were completely depolarised by less than 10 µg/ml Nod2 (Figure 3b). Again, this activity was deficient when the 3020insC LRR domain was used. Since LRR domains of Nod1 could also directly bind to bacteria (Figure 1c), we hypothesised that the observed anti-bacterial activity for Nod2 might be a common property of LRR domains derived from pattern-recognition receptors. LRR domains from Nod1, Naip, Nalp3, CIITA and TLR2 were generated and their activity against *B.subtilis* assessed in the membrane depolarisation assay (Figure 3c). Nod1 demonstrated comparable efficacy against the bacteria as Nod2. Naip, Nalp3 and TLR2 LRR domains all demonstrated significant activity in this assay – although this was not as robust as with Nod2 and Nod1 against *B.subtilis*.

We further extended our analysis of Nod2 anti-bacterial activity using a variety of established anti-bacterial assays to confirm our initial observations. In an agar-diffusion assay anti-bacterial activity of Nod2 could be observed with a clear zone of growth exclusion of *B.subtilis* and *S. aureus* surrounding a well containing purified LRR domains (Figure 3c). We did not observe any evidence of anti-bacterial activity using LRR proteins carrying the 3020insC mutation while Nod1 LRR domains also demonstrated a clear efficacy in this assay. Anti-bacterial activity was also tested using an *in vitro* assay based on the correlation of ATP levels with bacterial number (Table S2). Comparing Nod2 and Nod1 anti-bacterial activity in this assay demonstrated clear differences in bacterial specificity. For example, Nod2 effectively targeted *E. faecalis* while Nod1 was selective over Nod2 for *K. pneumoniae*. Not all bacteria were insensitive to the 3020insC LRR domain. Indeed, the activity of Nod2 and Nod2 3020insC was comparable against *L. monocytogenes*. This assay was also used to investigate the anti-bacterial activity of the Crohn's-associated G908R Nod2 polymorphism. *L. monocytogenes* continued to proliferate in the presence of the G908R LRR domain but was sensitive to the 3020insC domains. However, the anti-bacterial activity was not completely deficient in the G908R protein since it targeted *B. subtilis* with comparable efficacy to the 3020insC LRR (although this activity was deficient compared to the wild-type LRR domains).

The most pronounced observation was the clear sensitivity of the aerobic gram-positive bacteria tested in this assay to the Nod2 LRR domain. This was not observed when anaerobic bacteria were tested for LRR sensitivity in a standard minimal inhibitory concentration (MIC) assay. This assay assesses the potency of antibiotics by determining the minimal concentration at which no visible bacterial growth can be observed. The sensitivity of 9 anaerobic bacterial strains to Nod2 and Nod1 LRR domains using this assessment are presented in Table 1. All bacteria were insensitive to the presence of LRR domains containing the 3020insC mutation. On a weight basis, Nod1 and Nod2 efficacy was comparable with a standard broad-range antibiotic (Ciprofloxacin). On a molar basis, Nod1 and Nod2 were potent against the majority of anaerobic bacteria tested with Nod2 LRR MICs ranging from approximately 30 nM (*P. anaerobius*) to 4 µM (*B. thetaiotamicron*). This establishes the Nod proteins as among the most potent endogenous anti-microbial peptides yet identified [15].

**Table 1:** LRR domain anti-bacterial activity against anaerobes.

	Nod1 LRR	Nod2 LRR	3020insC LRR	Ciprofloxacin
*B.fragilis*	16-32 (4)	4-32 (5)	>128 (3)	8 (2)
*B.thetaiotamicron*	64-128 (3)	32-128 (4)	>128 (3)	64 (1)
*F.nucleatum*	16-64 (2)	8->128 (3)	>128 (2)	2 (1)
*P.intermedia*	8 (1)	4-8 (2)	>128 (1)	1 (1)
*E.lentum*	16 (1)	8-16 (2)	>128 (1)	2 (1)
*C.perfringens*	16-32 (3)	4-32 (4)	>128 (3)	2 (2)
*C.difficile* *	16-32 (3)	4-32 (5)	128->128 (3)	8 (3)
*P.anaerobius*	2 (1)	1-2 (2)	>128 (1)	1 (1)
*P.acnes*	8 (1)	4-8 (2)	>128 (1)	1 (1)

Minimal Inhibitory Concentrations µg/ml [lowest – highest (n)],

*data is composite of testing against three different strains.

## Discussion

These results have major implications for the pathogenesis of Crohn's disease. The compromised anti-bacterial activity demonstrated by the Crohn's-associated LRR domains should be considered alongside the signalling properties of the protein in any model of the molecular mechanisms associated with Nod2's role in Crohn's disease. Normal physiological events such as stress are associated with the onset of relapse in IBD patients and have been demonstrated to increase epithelial barrier permeability resulting in elevated levels of intracellular bacteria in normal animals [16],[17]. Breech of the epithelial barrier by bacteria would necessitate a response by the host to the invading microbe and Nod2 has a recognised role in coordinating innate immune responses to intracellular bacteria. In the context of Nod2 SNPs, the data presented would support the hypothesis that initial recognition and potential lysis of the intracellular bacteria by Nod2 is deficient in genetically defined patient populations. It is conceivable that this could lead to persistent infection of the host by the bacteria. A robust immune response would then be initiated by redundant bacterial-sensing pathways in the host to contain and deal with the infection. This could account for the relapsing, remitting and focal character of the inflammation observed in Crohn's disease patients. Inflammatory responses are essential to maintaining a barrier between the host and its environment. The direct recognition of bacteria by Nod2 likely triggers a cellular inflammatory response to breech of host defences by potential pathogens. While polymorphisms in the Nod2 LRR domains confer sensitivity to developing Crohn's disease, mutations in the Nod2 NACHT domain are associated with other inflammatory disorders, such as Early-Onset Sarcoidosis and Blau syndromes [18],[19]. This leads us to speculate that the Nod2 Nacht domain mutations may mimic in part an association of the protein with bacteria resulting in an elevated basal cellular anti-bacterial signaling response (in the absence of a bacterial infection). The Nod2 Nacht domain mutations are associated with eye, joint and skin, but not intestinal inflammation whereas the Crohn's-associated LRR domain polymorphisms are generally associated with intestinal, but not with extraintestinal, manifestations. Our data suggest that this may be due to a functional anti-bacterial LRR domain in the Nacht domain mutants suppressing any initiating bacterial infection in the gut that might lead to a Crohn's-like phenotype.

The discovery of a direct anti-microbial activity for Nod2 and Nod1 offers an explanation for the protection against bacterial infection conferred by expression of these proteins in cell lines and is consistent with the increased sensitivity of Nod2 knockout mice to *Mycobacteria, Listeria* and *Salmonella* infection [10]–[13], [20]. These data also have implications for infectious diseases associated with other LRR domain-containing proteins. Using *B. subtilis* as a model organism, we observed anti-bacterial activity using LRR domains derived from Nalp3, NAIP and TLR2 indicating that the LRR domain is a common anti-bacterial motif. Significant effects on *Listeria monocytogenes* were also observed with Nalp3 and TLR2 LRR domains using an ATP-based assay supporting this conclusion (Figure S4). Distinct categories of bacteria demonstrated sensitivity to different LRR domains suggesting that the LRR domains of individual PRRs have evolved to provide resistance to potential infection by specific pathogens. While many bacteria may be targeted directly by the LRR domains of the proteins that were tested, obvious candidates for future investigation are *Mycobacteria* for Nod2, *Legionella pneumophila* for NAIP and *Mycobacterium leprae* for TLR2 due to the evidence associating these proteins with susceptibility to infection in cells, animal models and their genetic links with patient populations [13],[21]–[26]. Nod2 deficiency in mice is associated with susceptibility to *Mycobacterial* infection [13]; a genus we did not consider in our study but one that has been debated as a candidate pathogen for Crohn's disease [27]–[29]. Nod2 SNPs associated with *Mycobacterial* infections are in general distinct from those observed in Crohn's disease or the Crohn's disease SNPs confer protection against *Mycobacterial*-dependent diseases [23],[24]. In addition, the Nod2-activating muramyl dipeptide motif in the bacterial proteoglycan coat is absent in *Mycobacteria*, although this does not preclude interaction of Nod2 with other PAMPs present on *Mycobacteria* [25]. Other SNPs identified in the leprosy, tuberculosis and Crohn's-susceptibility studies have identified common genes including IRGM, LRRK2 and TNFSF15 suggesting that a theme of host microbial defence underlies the pathogenesis of these diseases [23],[24],[30],[31].

Leucine-rich repeats are a conserved domain used in PRRs in most organisms and direct anti-bacterial activity may also play a significant role in innate defence against pathogens in plants via the R proteins [32]. Furthermore, agnathan fish possess an adaptive immune system based entirely on clonal rearrangement of LRRs rather than immunoglobulins and the resulting LRR domains demonstrate specificity for individual strains of bacteria [33]–[36]. Our data would predict that at least some of these LRR domains confer resistance to bacterial (and other microorganisms) infection by direct interaction and termination of the pathogen. This potential may underlie the conserved evolutionary development of the LRR domain as a PRR-associated motif.

In conclusion, our study has revealed a previously unappreciated anti-microbial activity for the LRR domains in a range of PRRs. Indeed, the clear association of Nod2 polymorphisms with defective bacterial killing suggests this function could significantly underlie the contribution of commensal flora to the pathogenesis of Crohn's disease. In addition, the direct anti-microbial effects demonstrated with other LRR domains suggest that direct recognition and termination of pathogens underlies the association of PRRs with susceptibility to infectious diseases and offers a rationale for PRR links with the development of autoimmune diseases. Efforts are now required to understand the role of Nod2 anti-microbial activity *in vivo* and reconcile this with known inflammatory signalling pathways.

## Materials and Methods

### DNA cloning

The LRR domains of Nod1, Nod2 and Nod2 3020insC, Nalp3, NAIP, CIITA and TLR2 were generated by PCR using primers flanking the LRR domain. The PCR fragments encompassed nucleotides 2275 to 3124 of Nod2 (NM_022162), nt2165 to nt3286 of Nod1 (NM_006092), nt2856 to nt3857 of Nalp3 (NM_004895), nt3692 to nt4930 of NAIP (NM_004536), nt2204 to nt3526 of CIITA (NM_000246) and nt58 to nt1983 of TLR2 (NM_003264). The integrity of all inserted fragments were confirmed by DNA sequencing.

### Protein purification

The LRR domains of Nod1, Nod2 and Nod2 3020insC, Nalp3, NAIP, CIITA and TLR2 were cloned in pDEST17 (Invitrogen) expressed in *Escherichia coli* Rossetta (DE3) cells (Novagen) and purified from inclusion bodies. The bacteria pellet were solubilized using Guanidine-HCl 6M and affinity purified by chromatography on a Ni–NTA column. Protein refolding was performed by a fast 10 fold dilution in PBS. A final purification step was performed using a HiLoad 16/60 Superdex 200 size-exclusion column. Purified proteins were visualized by Coomassie blue staining. The LRR domains were generally unstable, aggregated and demonstrated significant degradation upon storage. Nevertheless, comparable antibacterial activity was consistently demonstrated with over 20 individual protein preps.

### Antibodies

Anti-Nod2 rabbit antisera recognising recombinant human LRR domains purified from bacteria were generated by Eurogentec. Antibodies recognising the antigen were purified from the serum using a Nod2 LRR domain protein column and passed through a bacterial affinity column (Pierce) to remove contaminating anti-bacterial antibodies.

### *In vitro* anti-microbial assays

Bacterial viability was evaluated by standard MIC assay protocol in accordance with NCCLS standards [37]. The BacTiter-Glo microbial assay (Promega) was performed following the manufacturers instruction. Bacterial membrane depolarisation was assessed using a variation of the procedure previously described by Nuding and colleagues [14]. Agar diffusion assay was performed by placing purified protein in a well constructed in an agar plate containing an inoculated lawn of bacteria.

### High-resolution 1H-NMR

Cells were extracted with methanol/chloroform/water (1∶1∶0.9) as previously described [38]. High-resolution ^1^H-NMR spectra were acquired using standard pulse sequences at 300±1 K using a Bruker-600 Avance spectrometer (^1^H frequency of 600.26 MHz). ^1^H-NMR spectra were acquired with 256 FIDs, 65,536 complex data points, a spectral width of 7.2 kHz, and a relaxation delay of 1 s. Metabolite assignments were made on the basis of previous reported data and in certain cases confirmed by spiking [39].

## Supporting Information

Figure S1 Nod2 localisation of endogenous protein in SW480 intestinal epithelial cells in response to bacteria. SW480 cells were inoculated with *E.coli* (ATCC 25922) at an MOI of 1000∶1 as indicated and incubated for 2 hours. Cells were examined by immunofluorescence with αNod2 polyclonal antibody (generated and affinity purified as described in Figure 1) or rabbit IgG (processed over *E.coli* affinity column to remove *E.coli* interacting antibodies), FITC-conjugated phalloidin to detect actin and stained with DAPI to detect DNA (nucleii and bacterial DNA). Nod2 is distributed at low levels throughout the cytoplasm of unstimulated cells and colocalizes with intracellular bacteria following incubation with *E.coli*.
(0.20 MB PDF)

Figure S2 Immunofluorescent detection of recombinant Nod2 LRR domains with *E.faecalis*. Bacteria were incubated with either BSA (left panel) or Nod2 LRR domains (35 µg/ml; centre and right panels). Bacteria were processed and analysed using either anti-Nod2 antibody (left and centre panels) or without primary antibody (right panel) as controls. Insets demonstrate the presence of bacteria following staining with fluorescent membrane dye in each of the images. Bar = 50 µm.
(0.31 MB PDF)

Figure S3 Bacterial aggregation following incubation with recombinant Nod2 LRR domains. Bacteria were incubated overnight with 1 mg/ml (a,c,e) or 20 µg/ml (g) of BSA or equal concentrations of purified Nod2 LRR domains (b,d,f,h). a–f, Treated bacteria were incubated with membrane dye to help visualisation. Each sample was mixed by vortexing, a sample placed on a coverslip and observed by fluorescent microscopy. a,b, *Staphylococcus aureus*, c,d, *Streptococcus pneumoniae*, e,f, *Enterococcus faecalis*. g–h, *E.coli* were incubated as described above and visualised by phase microscopy.
(0.16 MB PDF)

Figure S4 TLR2 and Nalp3 LRR domains inhibit *L.monocytogenes* viability as demonstrated by ATP-coupled luminescent assay. *L.monocytogenes* (5X10^5^ bacteria/100 µl) were incubated with increasing concentrations of the indicated recombinant LRR domains for 6 hours at 37°C and ATP levels assessed by luminescent assay (BacTiter-glo: Promega). Values shown are relative to controls incubated in the absence of LRR domains (100%). Results are representative of two experiments for TLR2 and Nalp3. Recombinant LRR domains derived from CIITA and NAIP showed no activity in a single assay up to 50 µg/ml.
(0.05 MB PDF)

Table S1 Metabolite levels in treated *E. coli* (µmol/mg dry wt).
(0.03 MB DOC)

Table S2 LRR anti-bacterial activity against aerobic bacteria.
(0.04 MB DOC)
