# Supplementary material for: Direct Bacterial Killing In Vitro by Recombinant Nod2 Is Compromised by Crohn's Disease-Associated Mutations
Source: PLoS One. 2010 Jun 1;5(6):e10915. doi: 10.1371/journal.pone.0010915 (PMC2879363; doi:10.1371/journal.pone.0010915)
Supplement: Figure S1 — Nod2 localisation of endogenous protein in SW480 intestinal epithelial cells in response to bacteria. SW480 cells were inoculated with E.coli (ATCC 25922) at an MOI of 1000∶1 as indicated and incubated for 2 hours. Cells were examined by immunofluorescence with αNod2 polyclonal antibody (generated and affinity purified as described in Figure 1) or rabbit IgG (processed over E.coli affinity column to remove E.coli interacting antibodies), FITC-conjugated phalloidin to detect actin and stained with DAPI to detect DNA (nucleii and bacterial DNA). Nod2 is distributed at low levels throughout the cytoplasm of unstimulated cells and colocalizes with intracellular bacteria following incubation with E.coli. (0.20 MB PDF) [file pone.0010915.s001.pdf]

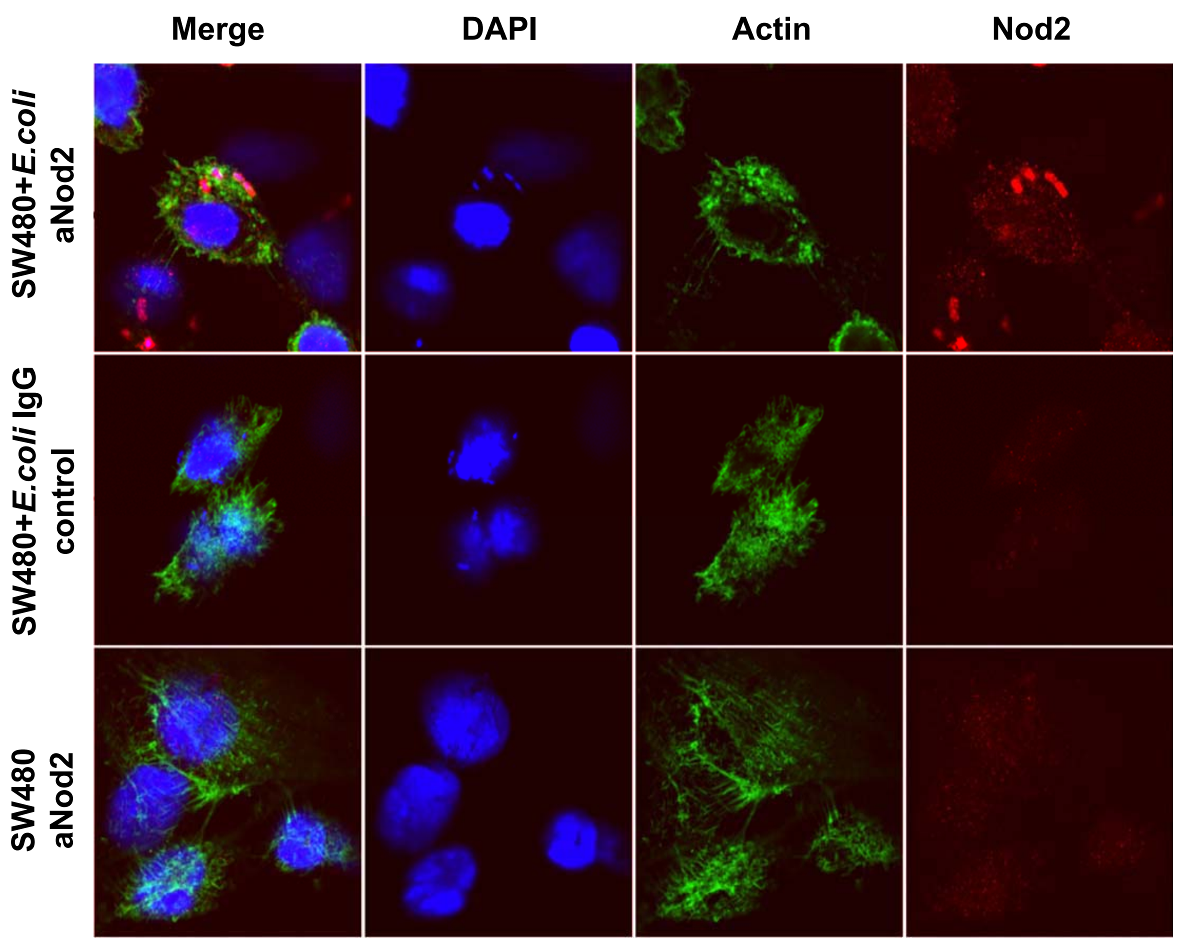

Supplementary Figure 1. Nod2 localisation of endogenous protein in SW480 intestinal epithelial cells in response to *E.coli*. SW480 cells were inoculated with *E.coli* (ATCC 25922) at an MOI of 1000:1 as indicated and incubated for 2 hours. Cells were examined by immunofluorescence with αNod2 polyclonal antibody (generated and affinity purified as described in Figure 1) or rabbit IgG (processed over *E.coli* affinity column to remove *E.coli* interacting antibodies), FITC-conjugated phalloidin to detect actin and stained with DAPI to detect DNA (nucleii and bacterial DNA). Nod2 is distributed at low levels throughout the cytoplasm of unstimulated cells and colocalizes with intracellular bacteria following incubation with *E.coli*.
