# Supplementary material for: Direct Bacterial Killing In Vitro by Recombinant Nod2 Is Compromised by Crohn's Disease-Associated Mutations
Source: PLoS One. 2010 Jun 1;5(6):e10915. doi: 10.1371/journal.pone.0010915 (PMC2879363; doi:10.1371/journal.pone.0010915)
Supplement: Figure S2 — Immunofluorescent detection of recombinant Nod2 LRR domains with E.faecalis. Bacteria were incubated with either BSA (left panel) or Nod2 LRR domains (35 µg/ml; centre and right panels). Bacteria were processed and analysed using either anti-Nod2 antibody (left and centre panels) or without primary antibody (right panel) as controls. Insets demonstrate the presence of bacteria following staining with fluorescent membrane dye in each of the images. Bar = 50 µm. (0.31 MB PDF) [file pone.0010915.s002.pdf]

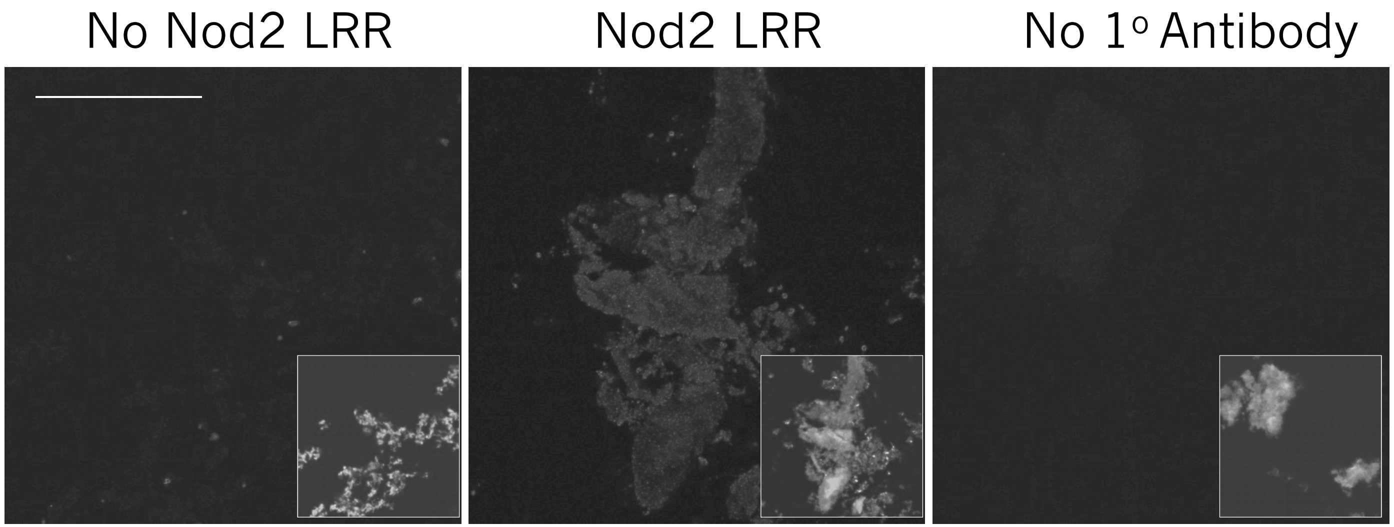

Supplementary Figure 2. Immunofluorescent detection of recombinant Nod2 LRR domains with *E.faecalis*. Bacteria were incubated with either BSA (left panel) or Nod2 LRR domains (35μg/ml; centre and right panels). Bacteria were processed and analysed using either anti-Nod2 antibody (left and centre panels) or without primary antibody (right panel) as controls. Insets demonstrate the presence of bacteria following staining with fluorescent membrane dye in each of the images. Bar = 50μm.
