# Supplementary material for: Direct Bacterial Killing In Vitro by Recombinant Nod2 Is Compromised by Crohn's Disease-Associated Mutations
Source: PLoS One. 2010 Jun 1;5(6):e10915. doi: 10.1371/journal.pone.0010915 (PMC2879363; doi:10.1371/journal.pone.0010915)
Supplement: Figure S3 — Bacterial aggregation following incubation with recombinant Nod2 LRR domains. Bacteria were incubated overnight with 1 mg/ml (a,c,e) or 20 µg/ml (g) of BSA or equal concentrations of purified Nod2 LRR domains (b,d,f,h). a–f, Treated bacteria were incubated with membrane dye to help visualisation. Each sample was mixed by vortexing, a sample placed on a coverslip and observed by fluorescent microscopy. a,b, Staphylococcus aureus, c,d, Streptococcus pneumoniae, e,f, Enterococcus faecalis. g–h, E.coli were incubated as described above and visualised by phase microscopy. (0.16 MB PDF) [file pone.0010915.s003.pdf]

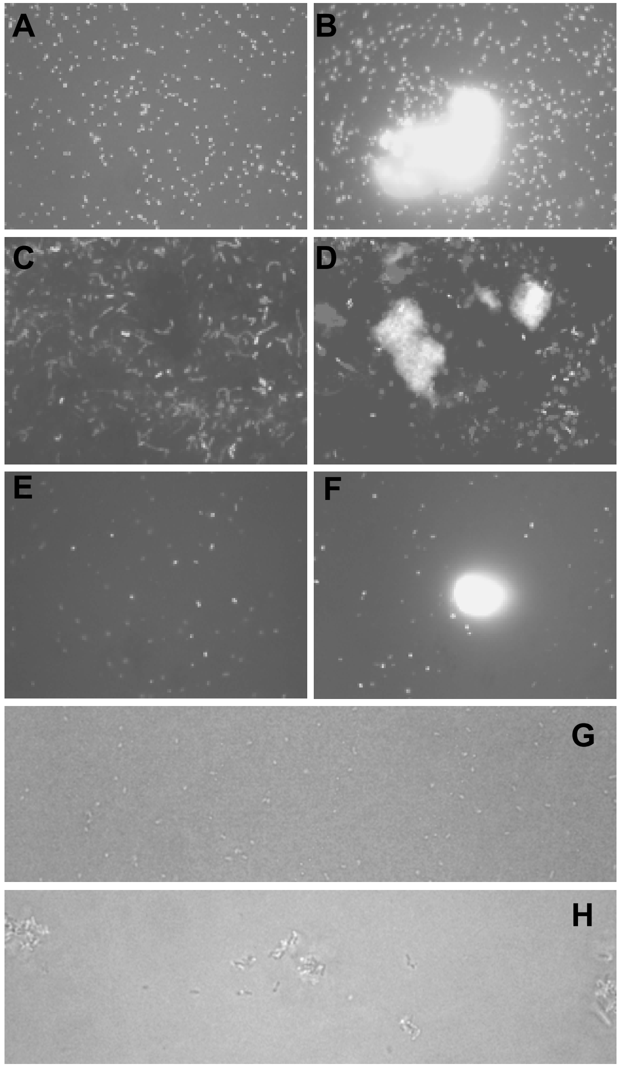

Supplementary Figure 3. Bacterial aggregation following incubation with recombinant Nod2 LRR domains. Bacteria were incubated overnight with 1mg/ml (a,c,e) or 20μg/ml (g) of BSA or equal concentrations of purified Nod2 LRR domains (b,d,f,h). **A-F**, Treated bacteria were incubated with membrane dye to help visualisation. Each sample was mixed by vortexing, a sample placed on a coverslip and observed by fluorescent microscopy. **A,B**, *Staphylococcus aureus*, **C,D,** *Streptococcus pneumoniae*, **E,F,** *Enterococcus faecalis*. **G,H**, *E.coli* were incubated as described above and visualised by phase microscopy.
