# Supplementary material for: Direct Bacterial Killing In Vitro by Recombinant Nod2 Is Compromised by Crohn's Disease-Associated Mutations
Source: PLoS One. 2010 Jun 1;5(6):e10915. doi: 10.1371/journal.pone.0010915 (PMC2879363; doi:10.1371/journal.pone.0010915)
Supplement: Figure S4 — TLR2 and Nalp3 LRR domains inhibit L.monocytogenes viability as demonstrated by ATP-coupled luminescent assay. L.monocytogenes (5X105 bacteria/100 µl) were incubated with increasing concentrations of the indicated recombinant LRR domains for 6 hours at 37°C and ATP levels assessed by luminescent assay (BacTiter-glo: Promega). Values shown are relative to controls incubated in the absence of LRR domains (100%). Results are representative of two experiments for TLR2 and Nalp3. Recombinant LRR domains derived from CIITA and NAIP showed no activity in a single assay up to 50 µg/ml. (0.05 MB PDF) [file pone.0010915.s004.pdf]

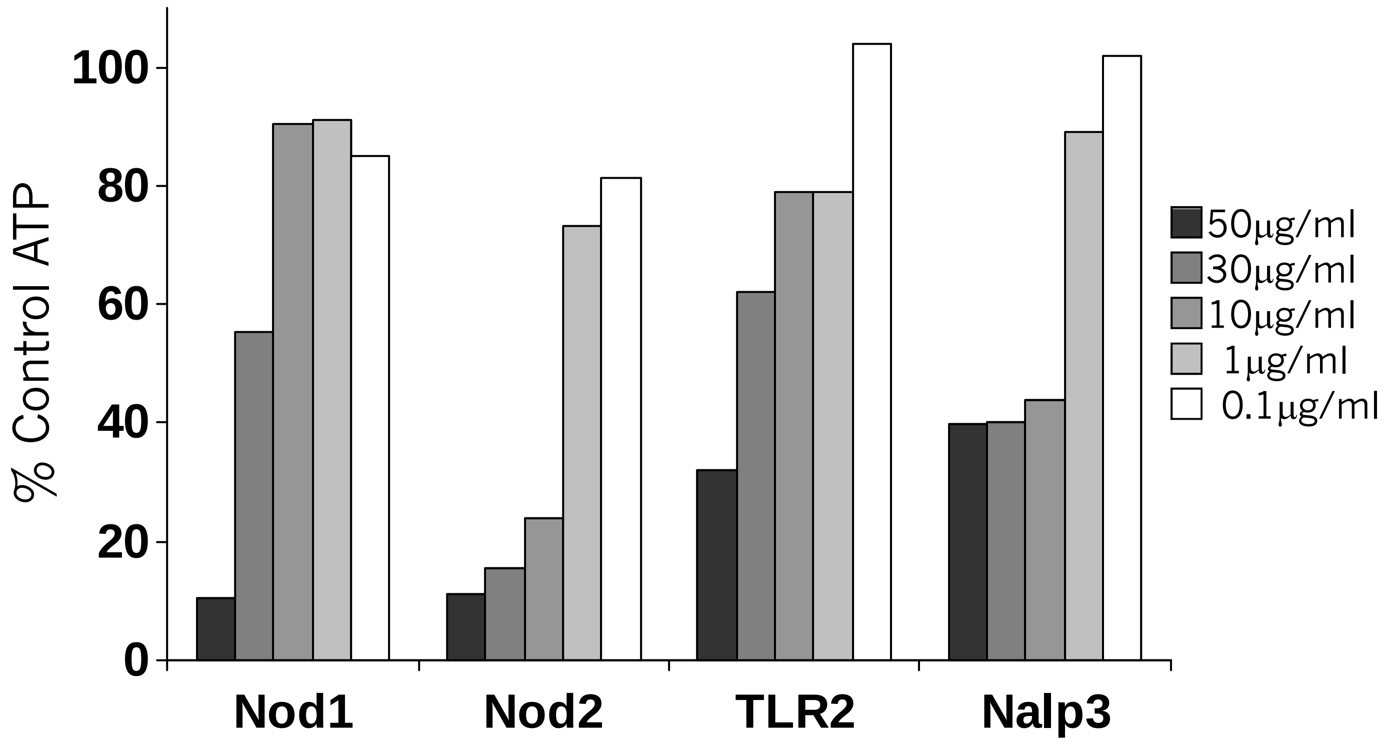

Supplementary Figure 4. TLR2 and Nalp3 LRR domains inhibit *L.monocytogenes* viability as demonstrated by ATP-coupled luminescent assay. *L.monocytogenes* ($5X10^5$ bacteria/100μl) were incubated with increasing concentrations of the indicated recombinant LRR domains for 6 hours at 37°C and ATP levels assessed by luminescent assay (BacTiter-glo: Promega). Values shown are relative to controls incubated in the absence of LRR domains (100%). Results are representative of two experiments for TLR2 and Nalp3. Recombinant LRR domains derived from CIITA and NAIP showed no activity in a single assay up to 50μg/ml.
