# Supplementary material for: Direct Bacterial Killing In Vitro by Recombinant Nod2 Is Compromised by Crohn's Disease-Associated Mutations
Source: PLoS One. 2010 Jun 1;5(6):e10915. doi: 10.1371/journal.pone.0010915 (PMC2879363; doi:10.1371/journal.pone.0010915)
Supplement: Table S1 — Metabolite levels in treated E. coli (µmol/mg dry wt). (0.03 MB DOC) [file pone.0010915.s005.doc]

Table S1: Metabolite levels in treated *E. coli* (μmol/mg dry wt).

| **Metabolite** | **Control** | **3020insC LRR** | **Nod2 LRR** |
| --- | --- | --- | --- |
| γ-aminobutyrate | 0.12±0.024 | 0.23±0.026 | 0.36±0.044^**†^ |
| Glutamate | 0.86±0.13 | 0.92±0.16 | 0.50±0.031^*†^ |
| Aspartate | 0.23±0.034 | 0.25±0.046 | 0.11±0.010^*†^ |
| Glutathione | 0.52±0.0086 | 0.53±0.011 | 0.25±0.0031^*†^ |
| Pyruvate | 0.56±0.13 | 0.67±0.14 | 0.92±0.069^*^ |
| TMAO | 4.1±0.65 | 4.8±0.89 | 8.5±0.59^**†^ |
| Tyrosine | 0.017±0.0025 | 0.022±0.0048 | 0.042±0.0031^**†^ |

Values are means ± Standard Deviation (n=4). *P≤0.05 versus Control. **P≤0.01 versus Control. ^†^P≤0.05 versus 3020insC.
