# Supplementary material for: Direct Bacterial Killing In Vitro by Recombinant Nod2 Is Compromised by Crohn's Disease-Associated Mutations
Source: PLoS One. 2010 Jun 1;5(6):e10915. doi: 10.1371/journal.pone.0010915 (PMC2879363; doi:10.1371/journal.pone.0010915)
Supplement: Table S2 — LRR anti-bacterial activity against aerobic bacteria. (0.04 MB DOC) [file pone.0010915.s006.doc]

Table S2: LRR anti-bacterial activity against aerobic bacteria.

| **Bacteria (ATCC)** | **Nod2** | **Nod2 3020insC** | **Nod2 G908R** | **Nod1** |
| --- | --- | --- | --- | --- |
| *L.monocytogenes (7644)* | 13.7+13.0 | 16.5+13.3 | None | 32.0+2.1 |
| *B.subtilis (6633)* | 3.9+0.6 | 54.0+20.2 | 45.4+2.0 | 15.5+12.0 |
| *E.faecalis (29212)* | 6.8+1.6 | None | None | >100 |
| *S.aureus (29213)* | 6.0+4.0 | None | None | 111.3+13.0 |
| *S.pneumoniae (49619)* | 3.0+1.6 | None | None | 13.5+4.9 |
| *E.coli (8739)* | >100 | None | None | 29.0+2.8 |
| *E.coli (25922)* | >100 | None | None | >100 |
| *K.pneumoniae (700603)* | None | None | NT | 30.8+4.9 |
| *P.aeruginosa (27853)* | None | None | None | >100 |
| *S.choleraesuis (13076)* | None | None | None | >100 |
| *S.maltophilia (17666)* | None | None | NT | >100 |

Growth assessed by luminescent reporter assay (BacTiter-glo: Promega) measuring ATP derived from bacteria grown at 37^o^C. Values are IC50 + Standard Deviation (n=3 independent experiments in duplicate). NT; not tested, >100; significant (but less than 50% decrease) in quantified ATP using 100μg/ml protein, None; no observed effect.
